# Artificial Placental Respiration

**Published:** 1872-12

**Authors:** J. N. Hyde

**Affiliations:** Secretary Chicago Society of Physicians and Surgeons; Chicago


ARTICLE VII.—*Artificial Placental Respiration.* By J. N. HYDE, M.D., Secretary Chicago Society of Physicians and Surgeons.

At a meeting of the Chicago society of Physicians and Surgeons, held November 12, 1872, a paper, which was avowedly a suggestion only, was read by Dr. John Bartlett, of Chicago, entitled, "*Artificial Placental Respiration.*"

Dr. Bartlett suggested in cases of extraction or extrusion of the after-birth before the child, the following practice: *Directly after* the detachment of the placenta, immerse it in the fresh warm defibrinated blood of some animal, as the sheep. Change the blood as often as practicable and aerate it by means of oxygen gas.

Dr. Bartlett considered the statements going to show that the life of the fœtus was not momentarily dependent upon the purification of its blood, through contact with the maternal circulation, too numerous to be disregarded. Three classes of cases in which the fœtus had, for a considerable time, survived a separation from the maternal circulation, were cited. In one class, the child maintained life in the dead body of the mother. In a second, the fœtus survived separation from the mother, its relations with the after-birth *and membranes* being retained. In a third class, the child remained alive in utero, while the placenta was without the mother.

The circumstances and conditions under which the fœtus may, for a considerable time, continue its life after disruption of its placental relations to the mother, seemed to be such as left the after-birth in a situation suitable to the performance of its *branchial* functions.

Dr. B. proceeded to point out by what simple processes of absorption and exhalation the embryo, in the lower forms of vertebrata, and in the earliest stages of embryonic existence in man, was provided with nourishment. He stated that the relation of the placenta of the chick to the albumen of the egg—its immersion in a fluid through which it absorbed oxygen and exhaled carbonic acid—was exactly such as he proposed to establish between the fœtus and the blood in which the extruded placenta was to be immersed. In regard to the probabilities of the simple immersion of the placenta in arterial blood, proving adequate to the temporary respiration of the fœtus, the essayist called attention to the rapidity with which absorption took place through even the smallest abraded surfaces. The placenta presented fifty square inches of serous, and an equal extent of exposed vascular, surface, through which the necessary interchange of gases might take place. In reference to the anatomy of the placenta, the language of Owen was quoted: "The placental intercommunication is carried on by the contact of the fœtal capillaries with the maternal *extravasated* blood." In this expression Dr. Bartlett regarded his suggestion as foreshadowed.

Artificial placental respiration would prove available in those cases of placenta prævia, in which the after-birth is detached, whether by the efforts of the uterus, or the accoucheur. It might also offer an additional chance of resuscitation to the asphyxiated child, when the placenta is already delivered. On account of the difficulty that might be experienced in obtaining the blood of an inferior animal just when needed, Dr. Bartlett suggested that the addition of phosphate of soda and oxygen gas to water, might render it a substitute. In some cases, the blood escaping from the mother might be employed.

